# 2-[(3-Chloro-2-methyl­phen­yl)amino]­quinoline-3-carb­oxy­lic acid

**DOI:** 10.1107/S2414314626005705

**Published:** 2026-06-02

**Authors:** Yujie Zuo, Sihui Long

**Affiliations:** a School of Chemical Engineering and Pharmacy Wuhan Institute of Technology,Wuhan Hubei 430205 People’s Republic of China; University of Antofagasta, Chile

**Keywords:** synthon, hydrogen bond, acid–acid dimer, single crystal

## Abstract

The title compound, C_17_H_13_ClN_2_O_2_, is an analogue of fenamic acid-type non-steroidal anti-inflammatory drugs. It was prepared from 2-chloro­quinoline-3-carb­oxy­lic acid and 3-chloro-2-methyl­aniline *via* palladium-catalysed Buchwald–Hartwig cross-coupling to afford the methyl ester inter­mediate, followed by alkaline hydrolysis. The mol­ecule adopts a nearly planar conformation with a dihedral angle of 7.17 (5)° between the quinoline ring system and the substituted phenyl ring. Adjacent mol­ecules form centrosymmetric carb­oxy­lic acid dimers *via* pairwise O—H⋯O hydrogen bonds.

## Abstract 2

Single crystals of 2-[(3-chloro-2-methyl­phen­yl)amino]­quinoline-3-carb­oxy­lic acid were obtained from slow evaporation of an ethyl acetate solution. In the crystal, the mol­ecules pair up to form acid–acid homodimers.

## Graphical abstract

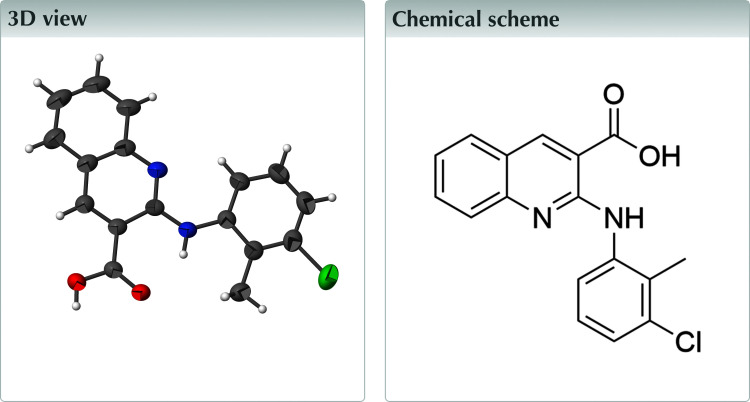

## Structure description

Non-steroidal anti-inflammatory drugs (NSAIDs) are mainstream clinical medicines with anti-inflammatory, analgesic and anti­pyretic activities. Fenamate-type di­aryl­amine derivatives serve as important lead scaffolds for anti-inflammatory drug discovery (Luan *et al.* 2017 ▸). However, traditional fenamate mol­ecules suffer from high conformational flexibility and disordered crystal packing, resulting in poor polymorphic stability and unstable pharmacological performance, which limits their clinical application (Uzoh *et al.* 2012 ▸). Replacing the benzene ring with a quinoline fused-ring moiety may enhance mol­ecular conjugation and planarity, thus optimizing the mol­ecular packing characteristics. To further explore the regulatory effects of substituents on mol­ecular structures and solid-state properties, the title quinoline-based fenamate derivative was constructed by introducing a 3-chloro-2-methyl disubstituted group onto the N-aryl ring. The substituent effects on mol­ecular conformations, hydrogen-bonding inter­actions and crystal packing were investigated, providing theoretical support for structural modification of this class of anti-inflammatory derivatives.

**Figure 1:**
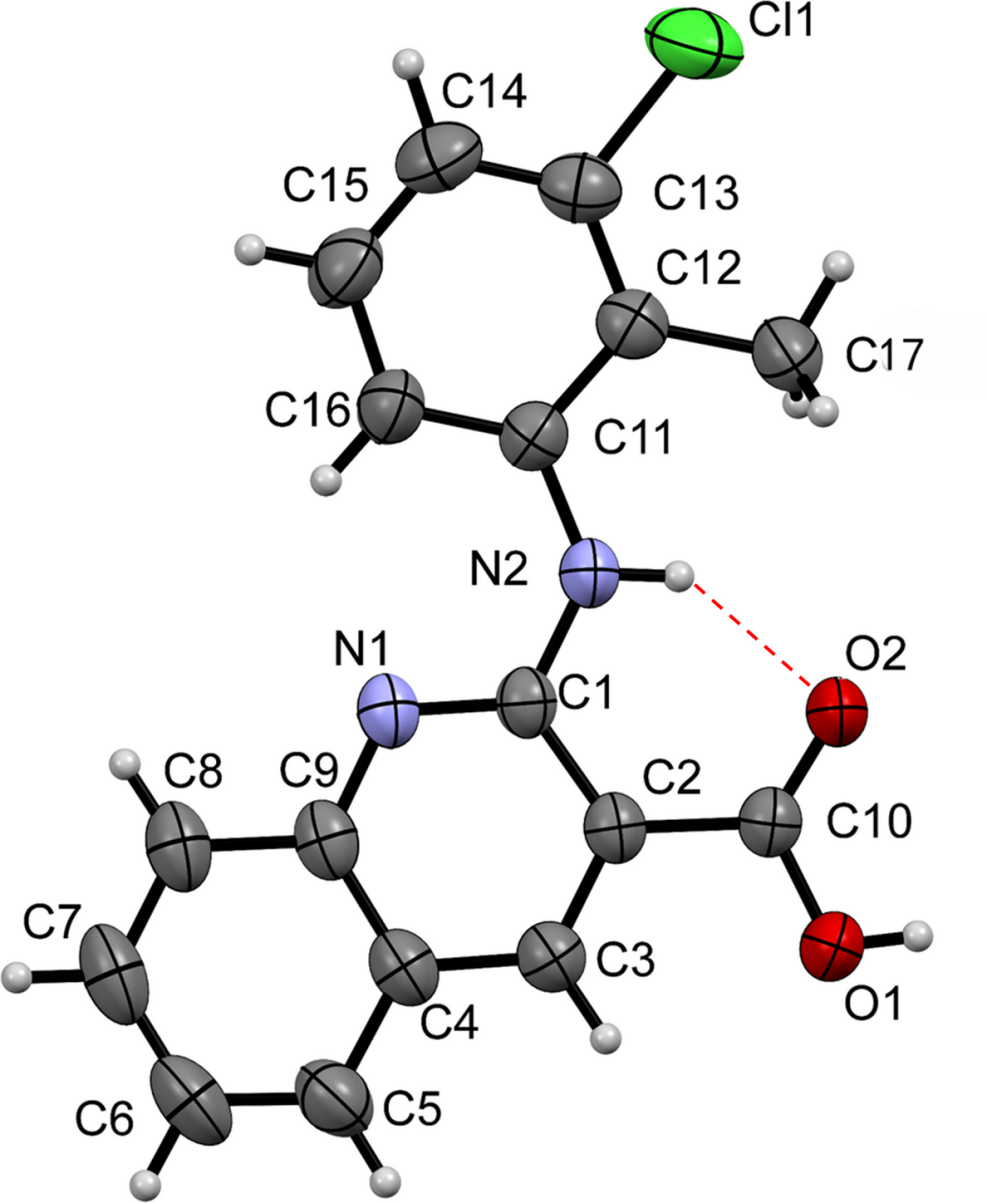
Mol­ecular structure of the title compound, with displacement ellipsoids drawn at the 50% probability level.

Herein, 2-[(3-chloro-2-methyl­phen­yl)amino]­quinoline-3-carb­oxy­lic acid (Fig. 1 ▸) was synthesized by a two-step route using 2-chloro­quinoline-3-carb­oxy­lic acid and 3-chloro-2-methyl­aniline as starting materials. applying the palladium-catalyzed Buchwald–Hartwig cross-coupling, followed by alkaline hydrolysis, acidification and purification (Janke *et al.* 2019 ▸). A strong intra­molecular N—H⋯O hydrogen bond is present in the mol­ecule (Table 1 ▸), with a donor–acceptor distance of 2.6919 (13) Å and a bond angle of 140°. This inter­action effectively restricts the free rotation of aromatic rings, yielding an approximately planar mol­ecular conformation with a dihedral angle of 7.17 (5)° between the quinoline ring system and the substituted benzene ring.

**Figure 2:**
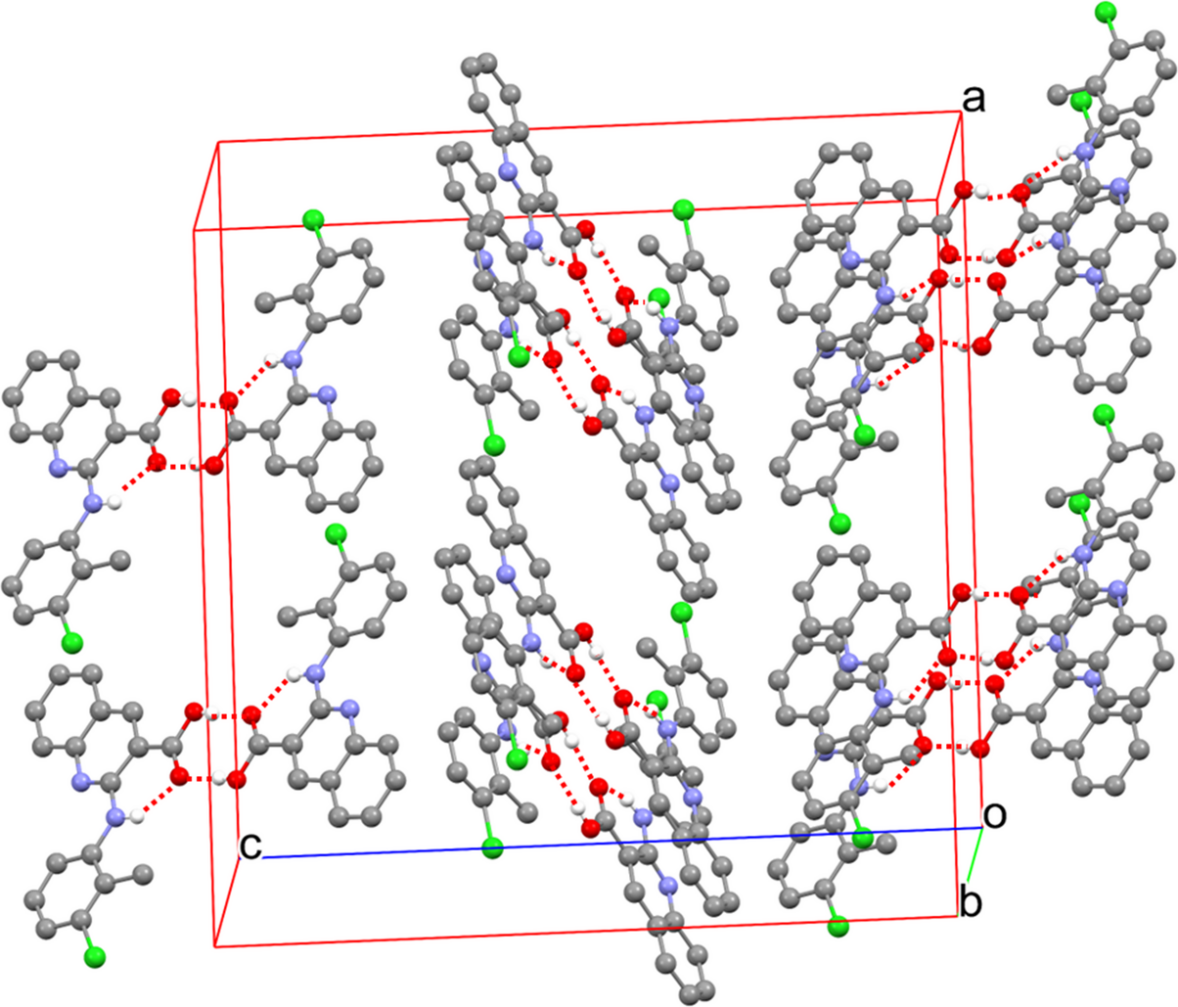
Packing of the mol­ecules in the title compound (for clarity, H atoms not involved in hydrogen bonding are omitted).

In the crystal, adjacent mol­ecules self-assemble into centrosymmetric carb­oxy­lic acid dimers through pairwise O—H⋯O hydrogen bonds. The donor–acceptor distance is 2.6532 (13) Å and the bond angle is 177°. These dimers further adopt a layered packing pattern (Fig. 2 ▸). The centroid-to-centroid distance between adjacent aromatic rings is 4.9749 (7) Å, hence no effective π–π stacking inter­actions are observed.

## Synthesis and crystallization

**Figure 3:**
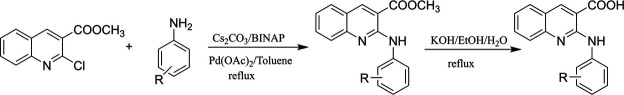
Synthesis of the title compound.

The title compound was synthesized in two steps using a Buchwald–Hartwig cross-coupling reaction followed by hydrolysis (Fig. 3 ▸). The compound was purified by column chromatography. Pale-yellow transparent block-shaped single crystals suitable for single-crystal X-ray diffraction measurements were grown by slow evaporation of an anhydrous ethyl acetate solution at ambient temperature.

## Refinement

Crystal data, data collection and structure refinement details are summarized in Table 2 ▸.

## Supplementary Material

Crystal structure: contains datablock(s) global, I. DOI: 10.1107/S2414314626005705/bx4041sup1.cif

Structure factors: contains datablock(s) I. DOI: 10.1107/S2414314626005705/bx4041Isup2.hkl

CCDC reference: 2558014

Additional supporting information:  crystallographic information; 3D view; checkCIF report

## Figures and Tables

**Table 1:** Hydrogen-bond geometry (Å, °)

*D*—H⋯*A*	*D*—H	H⋯*A*	*D*⋯*A*	*D*—H⋯*A*
O1—H1⋯O2^i^	0.82	1.83	2.6532 (13)	177
N2—H2⋯O2	0.86	1.97	2.6919 (13)	140

Symmetry code: (i) 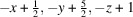.

**Table 2:** Experimental details

Crystal data	
Chemical formula	C_17_H_13_ClN_2_O_2_
*M*_r_	312.74
Crystal system, space group	Monoclinic, *C*2/*c*
Temperature (K)	298
*a*, *b*, *c* (Å)	27.6987 (4), 4.97483 (8), 24.2475 (4)
β (°)	97.6652 (14)
*V* (Å^3^)	3311.36 (9)
*Z*	8
Radiation type	Cu *K*α
μ (mm^−1^)	2.11
Crystal size (mm)	0.22 × 0.17 × 0.13

Data collection	
Diffractometer	XtaLAB Synergy R, DW system, HyPix
Absorption correction	Multi-scan (*CrysAlis PRO*; Rigaku OD, 2024 ▸)
*T*_min_, *T*_max_	0.747, 1.000
No. of measured, independent and observed [*I* > 2σ(*I*)] reflections	15744, 3303, 2939
*R*_int_	0.020
(sin θ/λ)_max_ (Å^−1^)	0.630

Refinement	
*R*[*F*^2^ > 2σ(*F*^2^)], *wR*(*F*^2^), *S*	0.038, 0.119, 1.08
No. of reflections	3303
No. of parameters	201
H-atom treatment	H-atom parameters constrained
Δρ_max_, Δρ_min_ (e Å^−3^)	0.17, −0.35

Computer programs: *CrysAlis PRO* (Rigaku OD, 2024 ▸), *SHELXT* (Sheldrick, 2015*a* ▸), *SHELXL2018/3* (Sheldrick, 2015*b* ▸) and *OLEX2* (Dolomanov *et al.*, 2009 ▸).

## full crystallographic data

### 2-[(3-Chloro-2-methylphenyl)amino]quinoline-3-carboxylic acid Crystal data

**Table:**

C_17_H_13_ClN_2_O_2_	*F*(000) = 1296
*M**_r_* = 312.74	*D*_x_ = 1.255 Mg m^−^^3^
Monoclinic, *C*2/*c*	Cu *K*α radiation, λ = 1.54184 Å
*a* = 27.6987 (4) Å	Cell parameters from 11991 reflections
*b* = 4.97483 (8) Å	θ = 3.7–75.3°
*c* = 24.2475 (4) Å	µ = 2.11 mm^−^^1^
β = 97.6652 (14)°	*T* = 298 K
*V* = 3311.36 (9) Å^3^	Block, clear light yellow
*Z* = 8	0.22 × 0.17 × 0.13 mm

### 2-[(3-Chloro-2-methylphenyl)amino]quinoline-3-carboxylic acid Data collection

**Table:**

XtaLAB Synergy R, DW system, HyPix diffractometer	3303 independent reflections
Radiation source: Rotating-anode X-ray tube, Rigaku (Cu) X-ray Source	2939 reflections with *I* > 2σ(*I*)
Mirror monochromator	*R*_int_ = 0.020
Detector resolution: 10.0000 pixels mm^-1^	θ_max_ = 76.4°, θ_min_ = 4.6°
ω scans	*h* = −34→34
Absorption correction: multi-scan (CrysAlisPro; Rigaku OD, 2024)	*k* = −6→6
*T*_min_ = 0.747, *T*_max_ = 1.000	*l* = −29→29
15744 measured reflections	

### 2-[(3-Chloro-2-methylphenyl)amino]quinoline-3-carboxylic acid Refinement

**Table:**

Refinement on *F*^2^	Primary atom site location: dual
Least-squares matrix: full	Hydrogen site location: inferred from neighbouring sites
*R*[*F*^2^ > 2σ(*F*^2^)] = 0.038	H-atom parameters constrained
*wR*(*F*^2^) = 0.119	*w* = 1/[σ^2^(*F*_o_^2^) + (0.0732*P*)^2^ + 0.6877*P*] where *P* = (*F*_o_^2^ + 2*F*_c_^2^)/3
*S* = 1.08	(Δ/σ)_max_ = 0.001
3303 reflections	Δρ_max_ = 0.17 e Å^−^^3^
201 parameters	Δρ_min_ = −0.35 e Å^−^^3^
0 restraints	

### 2-[(3-Chloro-2-methylphenyl)amino]quinoline-3-carboxylic acid Special details

**Table:**

Geometry. All esds (except the esd in the dihedral angle between two l.s. planes) are estimated using the full covariance matrix. The cell esds are taken into account individually in the estimation of esds in distances, angles and torsion angles; correlations between esds in cell parameters are only used when they are defined by crystal symmetry. An approximate (isotropic) treatment of cell esds is used for estimating esds involving l.s. planes.
Refinement. The position of the H atom in O and the position of the H atom in C are obtained from the differential Fourier diagram. The geometric positioning of the H atom is C—H = 0.93 for the aromatic group.

### 2-[(3-Chloro-2-methylphenyl)amino]quinoline-3-carboxylic acid Fractional atomic coordinates and isotropic or equivalent isotropic displacement parameters (Å^2^)

**Table:**

	*x*	*y*	*z*	*U*_iso_*/*U*_eq_	
Cl1	0.05724 (2)	0.47003 (12)	0.64179 (2)	0.08519 (19)	
O1	0.31240 (3)	1.13616 (19)	0.52322 (4)	0.0517 (2)	
H1	0.295888	1.243453	0.503101	0.078*	
O2	0.23926 (3)	1.00467 (18)	0.54053 (4)	0.0498 (2)	
N1	0.30894 (4)	0.4131 (2)	0.65151 (4)	0.0446 (2)	
N2	0.23512 (4)	0.5993 (2)	0.61329 (4)	0.0461 (3)	
H2	0.221813	0.709601	0.588480	0.055*	
C00H	0.13698 (5)	0.7379 (3)	0.58383 (6)	0.0592 (4)	
H00A	0.152806	0.905160	0.594516	0.089*	
H00B	0.102309	0.761483	0.580278	0.089*	
H00C	0.146033	0.680377	0.548826	0.089*	
C1	0.28489 (4)	0.5914 (2)	0.61842 (4)	0.0398 (3)	
C2	0.30970 (4)	0.7816 (2)	0.58646 (4)	0.0400 (3)	
C3	0.35948 (5)	0.7756 (3)	0.59269 (5)	0.0466 (3)	
H3	0.376165	0.895091	0.572572	0.056*	
C4	0.38588 (5)	0.5910 (3)	0.62913 (5)	0.0482 (3)	
C5	0.43749 (5)	0.5861 (3)	0.63887 (7)	0.0636 (4)	
H5	0.455480	0.704892	0.620105	0.076*	
C6	0.46074 (6)	0.4060 (4)	0.67600 (7)	0.0699 (4)	
H6	0.494581	0.404376	0.682981	0.084*	
C7	0.43373 (6)	0.2248 (3)	0.70336 (6)	0.0658 (4)	
H7	0.449972	0.101335	0.727994	0.079*	
C8	0.38385 (6)	0.2244 (3)	0.69483 (5)	0.0562 (3)	
H8	0.366614	0.101220	0.713457	0.067*	
C9	0.35855 (5)	0.4118 (2)	0.65761 (5)	0.0445 (3)	
C10	0.28369 (4)	0.9822 (2)	0.54854 (5)	0.0405 (3)	
C11	0.20208 (5)	0.4583 (2)	0.64160 (5)	0.0436 (3)	
C12	0.15239 (5)	0.5282 (3)	0.62760 (5)	0.0462 (3)	
C13	0.11928 (5)	0.3953 (3)	0.65606 (6)	0.0541 (3)	
C14	0.13227 (6)	0.2027 (3)	0.69635 (6)	0.0614 (4)	
H14	0.108869	0.119319	0.714622	0.074*	
C15	0.18075 (6)	0.1376 (3)	0.70868 (6)	0.0628 (4)	
H15	0.190216	0.007937	0.735593	0.075*	
C16	0.21570 (5)	0.2618 (3)	0.68171 (5)	0.0543 (3)	
H16	0.248298	0.214214	0.690344	0.065*	

### 2-[(3-Chloro-2-methylphenyl)amino]quinoline-3-carboxylic acid Atomic displacement parameters (Å^2^)

**Table:**

	*U*^11^	*U*^22^	*U*^33^	*U*^12^	*U*^13^	*U*^23^
Cl1	0.0481 (2)	0.1171 (4)	0.0934 (3)	0.0027 (2)	0.0205 (2)	0.0133 (3)
O1	0.0459 (5)	0.0534 (5)	0.0560 (5)	0.0025 (4)	0.0072 (4)	0.0183 (4)
O2	0.0420 (5)	0.0501 (5)	0.0562 (5)	0.0036 (4)	0.0028 (4)	0.0170 (4)
N1	0.0476 (6)	0.0440 (5)	0.0410 (5)	0.0062 (4)	0.0014 (4)	0.0040 (4)
N2	0.0419 (5)	0.0469 (6)	0.0485 (5)	0.0033 (4)	0.0023 (4)	0.0134 (4)
C00H	0.0480 (7)	0.0663 (9)	0.0632 (8)	0.0113 (6)	0.0071 (6)	0.0121 (7)
C1	0.0438 (6)	0.0381 (6)	0.0365 (5)	0.0030 (5)	0.0016 (4)	−0.0006 (4)
C2	0.0428 (6)	0.0384 (6)	0.0379 (5)	0.0037 (5)	0.0019 (4)	0.0000 (4)
C3	0.0438 (6)	0.0478 (7)	0.0480 (6)	0.0021 (5)	0.0050 (5)	0.0023 (5)
C4	0.0442 (7)	0.0505 (7)	0.0484 (6)	0.0084 (5)	0.0002 (5)	−0.0032 (5)
C5	0.0454 (7)	0.0704 (9)	0.0735 (9)	0.0082 (7)	0.0025 (6)	0.0019 (7)
C6	0.0471 (8)	0.0819 (11)	0.0765 (10)	0.0207 (7)	−0.0073 (7)	−0.0049 (8)
C7	0.0661 (9)	0.0672 (9)	0.0585 (8)	0.0262 (8)	−0.0125 (7)	−0.0020 (7)
C8	0.0630 (8)	0.0548 (7)	0.0478 (7)	0.0162 (6)	−0.0034 (6)	0.0019 (6)
C9	0.0497 (7)	0.0442 (6)	0.0376 (5)	0.0092 (5)	−0.0014 (5)	−0.0054 (5)
C10	0.0439 (6)	0.0383 (6)	0.0387 (5)	0.0015 (5)	0.0035 (5)	0.0012 (4)
C11	0.0465 (7)	0.0421 (6)	0.0419 (6)	−0.0016 (5)	0.0056 (5)	0.0008 (5)
C12	0.0475 (7)	0.0466 (6)	0.0448 (6)	0.0009 (5)	0.0074 (5)	−0.0032 (5)
C13	0.0490 (7)	0.0612 (8)	0.0534 (7)	−0.0026 (6)	0.0115 (5)	−0.0063 (6)
C14	0.0648 (9)	0.0666 (9)	0.0558 (8)	−0.0103 (7)	0.0189 (6)	0.0051 (7)
C15	0.0714 (9)	0.0633 (9)	0.0540 (7)	−0.0057 (7)	0.0090 (7)	0.0158 (7)
C16	0.0548 (7)	0.0549 (7)	0.0520 (7)	−0.0016 (6)	0.0029 (6)	0.0131 (6)

### 2-[(3-Chloro-2-methylphenyl)amino]quinoline-3-carboxylic acid Geometric parameters (Å, º)

**Table:**

Cl1—C13	1.7470 (15)	C4—C5	1.4175 (19)
O1—C10	1.3138 (14)	C4—C9	1.4088 (19)
O2—C10	1.2253 (15)	C5—C6	1.369 (2)
N1—C1	1.3158 (15)	C6—C7	1.395 (3)
N1—C9	1.3623 (16)	C7—C8	1.369 (2)
N2—C1	1.3682 (15)	C8—C9	1.4163 (17)
N2—C11	1.4029 (16)	C11—C12	1.4162 (18)
C00H—C12	1.5087 (18)	C11—C16	1.3952 (17)
C1—C2	1.4525 (16)	C12—C13	1.3867 (19)
C2—C3	1.3674 (17)	C13—C14	1.381 (2)
C2—C10	1.4777 (15)	C14—C15	1.375 (2)
C3—C4	1.4094 (18)	C15—C16	1.384 (2)
			
C1—N1—C9	119.40 (11)	N1—C9—C8	118.60 (12)
C1—N2—C11	131.02 (10)	C4—C9—C8	118.46 (12)
N1—C1—N2	119.80 (11)	O1—C10—C2	114.17 (10)
N1—C1—C2	121.82 (11)	O2—C10—O1	122.03 (10)
N2—C1—C2	118.38 (10)	O2—C10—C2	123.79 (11)
C1—C2—C10	123.09 (10)	N2—C11—C12	115.89 (11)
C3—C2—C1	117.96 (10)	C16—C11—N2	123.92 (12)
C3—C2—C10	118.95 (11)	C16—C11—C12	120.19 (12)
C2—C3—C4	120.97 (12)	C11—C12—C00H	120.85 (12)
C3—C4—C5	122.89 (13)	C13—C12—C00H	122.41 (12)
C9—C4—C3	116.87 (12)	C13—C12—C11	116.74 (12)
C9—C4—C5	120.22 (12)	C12—C13—Cl1	119.89 (11)
C6—C5—C4	119.76 (16)	C14—C13—Cl1	116.37 (11)
C5—C6—C7	120.05 (15)	C14—C13—C12	123.74 (13)
C8—C7—C6	121.58 (13)	C15—C14—C13	118.15 (13)
C7—C8—C9	119.91 (15)	C14—C15—C16	121.10 (13)
N1—C9—C4	122.94 (11)	C15—C16—C11	120.08 (13)

### 2-[(3-Chloro-2-methylphenyl)amino]quinoline-3-carboxylic acid Hydrogen-bond geometry (Å, º)

**Table:**

*D*—H···*A*	*D*—H	H···*A*	*D*···*A*	*D*—H···*A*
O1—H1···O2^i^	0.82	1.83	2.6532 (13)	177
N2—H2···O2	0.86	1.97	2.6919 (13)	140

Symmetry code: (i) −*x*+1/2, −*y*+5/2, −*z*+1.
